# Mortality among non-severely under nourished children with pneumonia globally: protocol for a systematic review and meta-analysis

**DOI:** 10.12688/wellcomeopenres.20200.2

**Published:** 2024-03-14

**Authors:** Damalie Nalwanga, Caitlin Bakker, Andrew Kiggwe, Abel Abera Negash, Moses Ocan, Andre Briend, Kathryn Maitland, Victor Musiiime, Charles Karamagi

**Affiliations:** 1 Department of Paediatrics and Child Health, School of Medicine, College of Health Sciences, Makerere University, Kampala, Central Region, Uganda; 2 Makerere University Lung Institute, Kampala, Central Region, Uganda; 3 University Libraries, University of Minnesota, Minneapolis, Minneapolis, USA; 4 Armauer Hansen Research Institute, Addis Ababa, Addis Ababa, Ethiopia; 5 Department of Microbiology, Immunology and Parasitology, School of Medicine, Addis Ababa University, Addis Ababa, Addis Ababa, Ethiopia; 6 Department of Pharmacology & Therapeutics, College of Health Sciences, Makerere University, Kampala, Central Region, Uganda; 7 School Of Medicine, University of Tampere, Tampere, Finland; 8 Department of Nutrition, Exercise and Sports, Faculty of Science, University of Copenhagen, Copenhagen, Capital Region of Denmark, Denmark; 9 Clinical, KERMI Wellcome Trust Research Programme, Kilifi, Kilifi, Po Box 230, Kenya; 10 Department of Infectious Disease and Institute of Global Health and Innovation, Imperial College London, London, England, UK; 11 Research Department, Joint Clinical Research Centre, Kampala, Uganda; 12 Clinical Epidemiology Unit, Department of Internal Medicine, School of Medicine, College of Health Sciences, Makerere University, Kampala, Central Region, Uganda

**Keywords:** Undernutrition, mortality, pneumonia, nutritional supplementation, children

## Abstract

**Background:**

Pneumonia remains the commonest cause of ill health and mortality among children worldwide. Severe undernutrition increases the mortality risk among children with pneumonia. While children with pneumonia are at increased risk of developing malnutrition, the impact of pneumonia on mortality and nutritional status of non-severely undernourished children is not well described. The impact of nutritional supplementation on mortality and nutritional status in this population is not well understood. This review will collate available evidence on the all-cause mortality and anthropometric indices outcomes following pneumonia, as well as the impact of nutritional supplementation on mortality and anthropometry among non-severely malnourished children with pneumonia.

**Methods:**

The review will be done using *a priori* criteria developed following the Preferred Reporting Items for Systematic Reviews and Meta-Analyses (PRISMA) guideline. Data will be obtained from data bases, grey literature, and bibliographies. An experienced librarian will conduct article search in PUBMED, MEDLINE, EMBASE, Web of Science, Google scholar, and Scopus. Retrieved articles will be entered in Endnote *ver* 9.0, duplicates removed, and transferred to Epi-reviewer for screening and data abstraction. Risk of bias in the included articles will be assessed using standard criteria. Heterogeneity will be assessed using I ^2^-statistic and sub-group analysis will be done. Data will be analysed using both narrative and quantitative synthesis. Quantitative synthesis will be done using DeSimonian and Laird Random-effects model in STATA *ver* 15.0.

**Conclusions:**

The results will provide baseline information about the mortality and anthropometric outcomes of pneumonia among non-severely malnourished children as well as the potential effect of nutritional supplementation on these outcomes. This will provide a basis to explore the potential for nutritional supplementation improving clinical outcomes like mortality and occurrence of severe acute malnutrition among children with severe pneumonia worldwide.

**Registration:**

The review has been registered in PROSPERO (CRD42021257272; 15 July 2021).

## Introduction

Worldwide, pneumonia remains the commonest cause of ill-heath and mortality among children under 5 years old ^1, 2^. Overall, 14% of all under-5 mortalities and 22% of mortalities among children 1-5 years are attributed to pneumonia, and majority occur in low- and middle-income countries (LMICs) where undernutrition is also most prevalent ^1– 4^. Mortality from pneumonia has widely been associated with and attributed to childhood undernutrition ^3, 5, 6^. The risk of severe pneumonia and mortality increases with the severity of undernutrition ^7– 9^. However, children with non-severe malnutrition and pneumonia are likely to progress to more severe forms of malnutrition, significantly increasing their risk of acquiring severe pneumonia and dying.

Undernutrition predisposes children to severe pneumonia *via* reduced immune responses towards infectious agents ^7, 9– 11^. Pneumonia causes a reduction in appetite, and often vomiting, as well as increase in energy demands required to support physiological changes like pyrexia and increased work of breathing ^12, 13^. As a result, children with pneumonia have an increased risk of developing undernutrition. This vicious cycle of pneumonia and undernutrition ^11, 14^ significantly increases the children’s risk of death, especially as undernutrition becomes more severe ^9, 15, 16^. Prevention of undernutrition through screening and timely intervention *e.g.*, through macronutrient supplementation in at-risk populations, such as children with pneumonia, could break the vicious cycle and significantly reduce pneumonia mortality.

The current treatment guidelines provide for identification and nutritional rehabilitation of children with severe acute malnutrition. However, there is no recommendation for nutritional supplementation among non-severely malnourished children with acute infections like pneumonia despite their imminent risk of developing severe forms of malnutrition. The World Health Organization (WHO) and UNICEF recommend “continued feeding” in their Protect, Prevent, and Treat framework for pneumonia management. However, there is no specific guidance on how feeding could be modified for better outcomes in the sick child, or whether nutritional supplementation could be beneficial ^17^. This could be due to scanty evidence supporting the need for nutritional support among non-severely malnourished children with pneumonia.

We propose a systematic review and meta-analysis to assess the all-cause mortality, as well as nutritional (anthropometry) outcomes of non-severely malnourished children with pneumonia, and to evaluate the impact of macronutrient supplement on mortality and anthropometry. This review will help to fill the information gap on the all-cause mortality among non-severely malnourished children with pneumonia, as well as compile available data on macronutrient supplement tested to reduce mortality and improve nutritional outcomes in this population. This information will provide evidence to utilize in developing guidelines or provide a basis for further studies to tackle the problem of pneumonia related mortality in this population.

## Protocol

### Study design

The review will be conducted following *a priori* criteria developed using the Preferred Reporting Items for Systematic Reviews and Meta-Analyses (PRISMA-P) guide ^18^. The review title has been registered in PROSPERO ( CRD42021257272; 15 July 2021) ^19^. The protocol has been reported according to the PRISMA-P guidelines ^20^.

### Research question

***Primary review question***

What is the all-cause mortality among non-severely malnourished children (2 months to 17 years old) with community acquired pneumonia globally?

***Secondary review questions***

- What is the mean change in anthropometric outcomes *i.e.*, mid-upper-arm-circumference (MUAC), Weight for Height z-score (WHZ), Weight for Age z-score (WAZ), BMI for age z score (BAZ) or Height for age z-score (HAZ) among non-severely malnourished children (2 months to 17 years) with pneumonia globally?
- What is the mean change in anthropometric status *i.e.*, MUAC, WHZ, WAZ, BMI for age z score (BAZ) and HAT among non-severely malnourished children (2 months to 17 years old) with community acquired pneumonia who received a macronutrient supplement compared to those who did not receive globally?
- What is the all-cause mortality among non-severely malnourished children (2 months to 17 years old) with community acquired pneumonia who received a macronutrient supplement compared to those who did not receive globally?

### Eligibility criteria

***Inclusion criteria***

- Articles published from 2000 to-date in peer-reviewed journals. This is because the WHO manual, which developed uniform ways for monitoring nutrition and diagnosis of malnutrition, was made in 1999 ^21^.
- Articles that report on mortality, and change in anthropometric status such as MUAC, WHZ, WAZ, or HAZ among non-severely malnourished children with pneumonia.
- Articles that report macronutrient supplementation such as ready to use therapeutic foods (RUTF) or enriched food given for any duration.

***Exclusion criteria***

- Articles from studies done exclusively among severely malnourished children, neonates (children under 1 month old) or older than 18 years old, and among children with hospital acquired pneumonia.

### Data sources

The data sources will include databases (PUBMED, Medical Literature Analysis and Retrieval System Online (MEDLINE), EMBASE, Web of Science, Cochrane Central, Google scholar, and Scopus), grey literature/bibliography, institutional websites and libraries, as well as published authors/ experts in the area.

### Data items

In this review, data will be sought on the following: community acquired pneumonia (CAP) among children ≥1 month ≤18 years old (population), non-severe malnutrition (exposure), mortality, change in anthropometric status (outcome), design of studies used to establish the all-cause mortality among non-severely malnourished children with pneumonia (study design), source of funding of the studies, and year when the studies were done (time period).

### Search strategy

The search for studies will be conducted systematically in databases including PUBMED, Medical Literature Analysis and Retrieval System Online (MEDLINE), EMBASE, Web of Science, Cochrane Central, Google scholar, and Scopus. The article search will be carried out by one re searcher (AK), and independently checked by another CB. The search terms will be combined using Boolean operators “AND” and “OR” for additive and restrictive combination of search terms as necessary. Websites of institutions that handle the population of interest will also be checked. The preliminary search in the PUBMED database is indicated in Table 1. The search string will be developed based on the population, intervention/exposure, Comparator, outcome (PICO), as summarised in Table 2. We shall also review the bibliographies of selected articles and contact lead researchers in the field for any additional potentially relevant article (s) from their research.

**Table 1.** Preliminary Search Strategy for PUBMED.

	PUBMED
1	Diet Therapy
2	Nutrition Therapy
3	undernourish* OR malnourish* OR poorly nourish* OR malnutrition
4	diet* OR diet therap* OR nutrition* OR nutrition* therap* OR nutrition* intervention* OR Nutrition* treatment* OR nutrition* status OR nutrition* modification OR nutrition*supplement*
5	macronutrient* OR calorie# OR caloric OR carbohydrate*intake OR protein* intake OR fat intake OR fat diet OR fats intake OR fats diet)
6	(diet OR food) AND nutrition
7	nutrition disorder* OR child nutrition disorder* OR infant nutrition disorder* OR malnutrition
8	"ready to use therapeutic food" OR "ready to use therapeutic food" OR "ready-to-use therapeutic food" OR "ready-to-use therapeutic food" OR RTUF OR food* supplement*
9	Dietary Carbohydrates OR Dietary Proteins OR Fats OR Fortified
10	Dietary supplements OR functional food
11	or/1-10
12	pneumonia
13	pneumonias OR pneumonic
14	12 or 13
15	Neonates OR premature infants OR infant OR newborn OR infant OR infant behavior OR child, preschool OR child behavior OR child development OR child psychiatry OR orthopsychiatry OR child psychology OR child behavior disorders OR pediatrics OR child OR puberty OR adolescent OR adolescent behavior OR adolescent development OR adolescent psychiatry OR adolescent psychology OR young adult OR schools, nursery OR child day care centers OR child care OR education, graduate OR universities OR students OR schools
16	preterm OR premature OR postmature OR perinatal OR postnatal OR neonatal OR newborn OR new-born OR infant OR baby OR babies OR toddler* OR preschool* OR child* OR pediatric OR paediatric OR kid OR kids OR prepubescent OR prepuberty OR puberty OR pubescen* OR teen* OR young* OR youth* OR minors OR under age OR underage OR juvenile* OR girl* OR boy* OR preadolesc* OR adolesc*
17	15 or 16
18	11 and 14 and 17
19	Mortality OR survival OR death
20	18 and 19
21	limit 20 to yr="2000 -Current" (what I sent later)-less study design filters
22	Exclude animals
23	Limit to Clinical Trials, multicentre trials, RCTs, pragmatic clinical trials and pragmatic clinical studies

**Table 2.** PICOS definitions.

Population	• Children ≥1 month ≤ 18 years • Community acquired Pneumonia (World Health Organization (WHO) definition), lower respiratory tract infection, respiratory tract infection • Globally • Hospitals (any level of health facility) • Sample size	• Age <1 month/ neonate, or adults/> 18 years • Hospital acquired pneumonia • Pneumonia among specific categories of patients e.g., intensive care unit (ICU) patients, cystic fibrosis, post-operative patients, sickle cell patients • Pneumonia diagnosed only by chest X-ray or culture and sensitivity, C-reactive protein (CRP) only
Exposure/Intervention for interventional studies	• Malnutrition or anthropometric status, underweight, undernourished, stunted • Any nutritional intervention; with food e.g., porridge, rice porridge, or other enriched food, ready to use therapeutic food (RUTF)	• Reports only on **severely malnourished** children • Micronutrient-only interventions *e.g.*, zinc, iron, selenium, Vitamins *etc*.
Outcome	• Mortality/death • Any change in mid upper arm circumference (MUAC), weight for height z scores (WHZ), body mass index (BMI), skinfold thickness, bio-impedance analysis (BIA)	• Any outcomes other than mortality and change in MUAC
Study Design	• Observational/ Cohort • Cross-sectional • Randomized controlled trial (RCT) • Survey • Interventional but non-randomized • Systematic Review	• Case report • Case series
Time Period	• Published after 1999 (2000 and beyond)	

### Article screening

Three pairs of independent reviewers (DN, LN, TK, GK, RA and MO) will screen the articles using pre-determined screening criteria, first using titles and abstracts, and then using the selected full texts. The selection of studies will be performed independently in pairs that will be blinded to each other’s decisions. After unblinding, the two reviewers will resolve any conflicts by consensus. A third reviewer will analyse and decide in cases where there are conflicting decisions but no consensus.

### Data extraction

The tool for data abstraction will be developed in Excel spreadsheet and will include sections on: general study information including the country research was done and year of data collection and publication; the study design, study population and comparators if any; anthropometric status and its assessment method, and intervention used if any; mortality proportions and anthropometric status outcome. The tool will be piloted in six ^6^ articles and the findings used to adjust the tool. The tool will then be used to develop an abstraction screen in Epi-Reviewer software. Data abstraction will be done by (DN, LN, TK, GK, RA and MO). The reviewers will be paired and any disagreements between any pair resolved through discussion and consensus. Further disagreement will be referred to a tie breaker. Authors will be contacted in cases where more information, or clarification is required.

### Quality assessment

Assessment of the quality of studies included will be done to evaluate bias and reliability of the evidence using standardized tools (The Risk of Bias in Non-randomised Studies - of Interventions (ROBINS-I) for non-randomized interventional studies, Cochrane risk-of-bias tool revised for randomized trials and the Risk of Bias in Non-randomised Studies (ROBINS) for observational studies). Two reviewers will independently carry out the validity assessment. Any disagreement between the reviewers will be resolved by discussion and consensus.

### Data management

The identified articles from the different databases will be imported into Endnote reference manager™ version X7 (Thomson Reuters, 2015). Articles will be screened for duplicates, which will be removed. The EPPI-Reviewer™ Software (UK) version 6 tool will be used for article screening (title and abstract), full text screening and data abstraction.

### Heterogeneity assessment

We shall measure heterogeneity among studies using the I ^2 ^statistic to estimate the percentage of variation among the studies included in the review. A heterogeneity will be considered low if the I ^2 ^statistic ranges between 0% to 40%, moderate if it ranges between 30% to 60%, substantial if it ranges between 50% to 90%, and high if it is 75% to 100% ^22, 23^.

### Data analysis and synthesis

Data will be analysed using both narrative and quantitative synthesis methods. Categorical data from all the individual studies will be summarized as frequencies and percentages and numerical continuous data as means and standard deviations or median and interquartile range for parametric and non-parametric data, respectively. Quantitative synthesis will be done using DeSimonian and Laird Random-effects model in STATA ver 18.0. For the calculation of pooled effect estimates we will consider a confidence interval (CI) = 95% and α = 0.05 using EPPI-Reviewer™ Software (UK). The results will be presented in PRISMA 2020 flow chart ^18^ and as forest plots and sub- group analyses will be carried out by participant age, duration of follow up, severity of pneumonia as specified in the selected articles. From heterogeneity analysis, we shall identify the articles to be included in quantitative analysis. For intervention studies that qualify for quantitative synthesis, further assessment of how categorical study characteristics are associated with the intervention effects will be done (meta-regression). A p-value of 0.05 will be considered statistically significant for all hypothesis testing.

### Publication bias assessment

Publication bias refers to how the research findings impact the chances that a study is published or not. We shall explore the probability of publication bias among the selected articles using funnel plots and assessing the symmetry of the plots. Adjustment for publication bias will be performed by the trim and fill method ^24^.

### Patient and public involvement

The study will include research articles and have no direct engagement of patients or members of the public.

### Ethics and dissemination

As there will be no direct involvement of human subjects, ethics approval is not applicable. We shall disseminate the research finding to clinicians and researchers through dissemination meetings, conferences, and in peer-reviewed publications. No sex and/or gender (which are based on self-reporting within the manuscripts reviewed) differences are expected in the results.

## Study status

The article search has been completed. Screening by title and abstract has also been completed. Screening by full text is ongoing.

## Discussion

This study aims to assess the all-cause mortality, as well as anthropometric outcomes among non-severely malnourished children with pneumonia, and to evaluate the impact of macronutrient supplementation on mortality and anthropometry.

Results from this study will help to document the all-cause mortality among non-severely malnourished children with pneumonia, as well as their anthropometric outcomes following pneumonia episodes. We also hope to document the effect of nutritional supplementation on mortality and anthropometric outcomes in this population. This will provide some baseline evidence on the need for nutritional rehabilitation among non-severely malnourished children with pneumonia in order to potentially reduce pneumonia mortality and improve anthropometric outcomes in this population.

### Strengths and limitations

***Strengths***

Restricting the review to the period when the WHO manual with uniform ways for monitoring nutrition and diagnosis of malnutrition in children was in effect will help generate comparable evidence on mortality among non-severely nourished children with pneumonia, and the effect of supplementary food on anthropometric outcomes in this population.

Including studies published globally will help in providing context to the findings of the review.

***Limitations***

The settings in which the primary studies were conducted, as well as the level of care given to the children may not be uniform, which could impact the findings from this study.

***Conclusion***

This review will collate available evidence on the all-cause mortality, anthropometric outcomes following pneumonia, as well as the impact of nutritional supplementation on mortality and anthropometry among non-severely malnourished children with pneumonia.

## Data Availability

No data are associated with this article. The protocol has been reported according to the PRISMA-P guidelines. Imperial College Research Data Repository: PRISMA checklist for ‘Mortality among non-severely under nourished children with pneumonia globally: protocol for a systematic review and meta-analysis’. https://doi.org/10.14469/hpc/13333 ^20^. Data are available under the terms of the Creative Commons Zero “No rights reserved” data waiver (CC0 1.0 Public domain dedication).
