# Supplementary material for: Mortality among non-severely under nourished children with pneumonia globally: protocol for a systematic review and meta-analysis
Source: Wellcome Open Res. 2024 Mar 14;8:551. Originally published 2023 Nov 28. [Version 2] doi: 10.12688/wellcomeopenres.20200.2 (PMC10891427; doi:10.12688/wellcomeopenres.20200.2)
Supplement: Peer review history

# Reviewer report: Reviewer response for version 2

**DOI:** 10.21956/wellcomeopenres.23401.r76795

**Published:** 2024-03-28

**Authors:** Felix Bongomin

**Affiliations:** 1 Gulu Unversity, Gulu, Uganda

The authors have addressed  all comments sufficiently.

Is the study design appropriate for the research question?

Yes

Is the rationale for, and objectives of, the study clearly described?

Yes

Are sufficient details of the methods provided to allow replication by others?

Partly

Are the datasets clearly presented in a useable and accessible format?

Yes

Reviewer Expertise:

Internal Medicine, Medical Microbiology and Research Methodology

I confirm that I have read this submission and believe that I have an appropriate level of expertise to confirm that it is of an acceptable scientific standard.

# Reviewer report: Reviewer response for version 1

**DOI:** 10.21956/wellcomeopenres.22363.r72532

**Published:** 2024-02-21

**Authors:** Olivier Marcy

**Affiliations:** 1 IRD EMR271, INSERM U1219, Bordeaux Population Health, University of Bordeaux, Bordeaux, France

This is an interesting, needed, and well written meta-analysis protocol on pneumonia and non-severe or moderate acute malnutrition (MAM) in children and adolescents, and particularly on the impact of MAM on pneumonia mortality and vice-versa of pneumonia on occurrence of severe acute malnutrition (SAM), as well as the effect of micronutrient on nutritional and vital outcomes. When there is a wealth of data on the impact of MAM/SAM on pneumonia mortality, impact of pneumonia on nutritional outcomes is much less documented. Micronutrient supplementation could play an important role in reducing severe outcomes and findings from this study could have important public health impact.

 I have 3 comments to consider if possible either at protocol stage or in the analysis.

1. 0 to 19 is quite a wide age range and MAM in children below the age of 5 years is quite different than in adolescents aged >10 as defined by the WHO or above 13-14. I would suggest stratifying the analysis by age group and having a strong focus on the age group 2 to 59 months, which bears the highest burden in terms of mortality.
2. I agree with using several malnutrition indicators and suggest adding BMI for age between -2 and -3 SD as an indicator for MAM in children above the age of 5.
3.  I suggest phrasing objective #2 as objective#3 is phrased and introducing the notion of comparison between groups rather than stating “due to macronutrient supplementation”.

Is the study design appropriate for the research question?

Yes

Is the rationale for, and objectives of, the study clearly described?

Yes

Are sufficient details of the methods provided to allow replication by others?

Yes

Are the datasets clearly presented in a useable and accessible format?

Not applicable

Reviewer Expertise:

Childhood tuberculosis and pneumonia including in children with severe acute malnutrition.

I confirm that I have read this submission and believe that I have an appropriate level of expertise to confirm that it is of an acceptable scientific standard.

# Reviewer report: Reviewer response for version 1

**DOI:** 10.21956/wellcomeopenres.22363.r71396

**Published:** 2024-02-01

**Authors:** Felix Bongomin

**Affiliations:** 1 Gulu Unversity, Gulu, Uganda

Nalwanga et al presents a well written protocol for a systematic review and meta-analysis of mortality among non-severely under nourished children with pneumonia globally following the PRISMA-P guidelines.

 Minor comments

 1. There is a repetitive use of "nutritional (anthropometric)" - authors could elect to use nutritional or anthropometric and apply it consistently through out the manuscript 

 2. PRISMA - P not PRISMA guidelines 

 3. Consider using later version of STATA , eg version 18

 4. Is it the search strategy or the research question that is developed using the PICO  criteria ?  I would think it's the research question and would advise the authors to revise these sections accordingly.

 5. Consider adding a sentence on meta-regression in the analysis section

 6. Discusion section lasts citation of other related studies and systematic reviews , which would then be used to compare findings from this study

 7. add a conclusion after the strengths and limitation section

Is the study design appropriate for the research question?

Yes

Is the rationale for, and objectives of, the study clearly described?

Yes

Are sufficient details of the methods provided to allow replication by others?

Partly

Are the datasets clearly presented in a useable and accessible format?

Yes

Reviewer Expertise:

Internal Medicine, Medical Microbiology and Research Methodology

I confirm that I have read this submission and believe that I have an appropriate level of expertise to confirm that it is of an acceptable scientific standard, however I have significant reservations, as outlined above.

# Reviewer report: Reviewer response for version 1

**DOI:** 10.21956/wellcomeopenres.22363.r72538

**Published:** 2024-01-27

**Authors:** Rouzha Pancheva

**Affiliations:** 1 Faculty of Public health,Department of Hygiene and Epidemiology, Medical University, Varna, Varna, Bulgaria

Comprehensiveness in Context of Current Literature: The article appears to thoroughly discuss the protocol for the topic of mortality among non-severely undernourished children with pneumonia. It references current treatment guidelines and the lack of recommendations for nutritional supplementation in non-severely malnourished children with pneumonia, indicating an awareness of the existing literature and practices. The proposed systematic review and meta-analysis aim to fill an information gap, suggesting a comprehensive approach.

 Factual Accuracy and Citations: The document contains numerous references to support its statements, ranging from WHO guidelines to various studies and systematic analyses. This suggests a strong basis in factual accuracy, supported by adequate citations.

 Accessible Language: The language used in the article is technical, reflecting its academic nature. However, it seems clear and precise, which is appropriate for its intended audience of clinicians and researchers. It might not be considered 'accessible' to a layperson due to the use of specialized terms and concepts, but for its target audience, it appears suitable.

 Appropriateness of Conclusions: The document primarily outlines the protocol for a systematic review and meta-analysis rather than presenting conclusions from these analyses. However, the rationale for the study, its objectives, and the methodology are clearly aligned with current research literature. The anticipated outcomes suggest that the study’s conclusions will likely be relevant and appropriate in the context of existing research.

 Overall, the document is well-structured and appears to meet the necessary criteria for a comprehensive, factually accurate, and appropriately targeted academic article.

Is the study design appropriate for the research question?

Yes

Is the rationale for, and objectives of, the study clearly described?

Yes

Are sufficient details of the methods provided to allow replication by others?

Yes

Are the datasets clearly presented in a useable and accessible format?

Yes

Reviewer Expertise:

Nutrition, child development, neurodevelopmental disabilities

I confirm that I have read this submission and believe that I have an appropriate level of expertise to confirm that it is of an acceptable scientific standard.
